# Medical Device Industry Payments to Healthcare Professionals and Organizations in Japan: An Evaluation of Scale, Distribution, and Transparency Practices, 2019-2022

**DOI:** 10.34172/ijhpm.9079

**Published:** 2026-06-01

**Authors:** Akemi Hara, Sunil Shrestha, Hiroaki Saito, James Larkin, Frank Moriarty, Piotr Ozieranski, Rajeev Shrestha, Tetsuya Tanimoto, Akihiko Ozaki

**Affiliations:** ^1^Medical Governance Research Institute, Tokyo, Japan.; ^2^Department of Research and Academics, Kathmandu Cancer Center, Bhaktapur, Nepal.; ^3^Department of Internal Medicine, Soma Central Hospital, Fukushima, Japan.; ^4^Department of General Practice, Royal College of Surgeons in Ireland, University of Medicine and Health Sciences, Dublin, Ireland.; ^5^Department of Social and Policy Sciences, University of Bath, Bath, UK.; ^6^NIHR Newcastle Patient Safety Research Collaboration, Newcastle University, Newcastle upon Tyne, UK.; ^7^Navitas Clinic Kawasaki, Kanagawa, Japan.; ^8^Breast and Thyroid Center, Jyoban Hospital of Tokiwa Foundation, Iwaki, Japan.

**Keywords:** Medical Devices, Conflict of Interest, Financial Disclosure, Transparency, Japan

## Abstract

**Background:**

Financial relationships between the medical device industry and healthcare providers have raised concerns about conflicts of interest (COIs). However, these relationships remain poorly characterized in Japan, despite the country’s large medical device market, which was valued at ¥4.41 trillion ($33.3 billion) as of 2021. This study examined the scale, composition, and temporal patterns of payments from the medical device industry and evaluated current transparency practices.

**Methods:**

We analyzed publicly disclosed payment data from 117 medical device companies predominantly affiliated with the Japan Federation of Medical Devices Associations (JFMDA) from 2019 to 2022. Payment categories included research and development, academic research support, lecture and consulting fees, information-provision–related expenses, and other payments to healthcare professionals (HCPs) and healthcare organizations (HCOs). We assessed payment magnitude, category composition, company-level concentration, year-to-year changes, and disclosure transparency using an adapted proforma previously applied to European pharmaceutical payment data.

**Results:**

Total payments amounted to $942.3 million over four years. Academic research support expenses constituted the largest share (33.0%, $310.7 million), followed by information provision–related expenses (25.2%, $237.5 million) and research and development expenses (21.7%, $204.8 million). Payments were highly concentrated, with the top 10 companies accounting for approximately 58% of total amounts. Using 2019 as the pre-pandemic baseline, total payments declined by 30.2% in 2020 and remained below pre-pandemic levels in 2021 (−24.0%), before partially recovering in 2022 (−11.6%). Category-specific trends diverged during the pandemic, with consulting, lecturing, and manuscript-related fees exceeding pre-pandemic levels by 2022, while information provision–related expenses remained substantially reduced. Transparency was limited: 78.6% of companies disclosed payment data with limited standardization, searchability, or data download functionality.

**Conclusion:**

This multi-year analysis revealed substantial financial relationships between the medical device industry and healthcare stakeholders in Japan, alongside persistent shortcomings in transparency of disclosures. Introducing legally mandated disclosure would improve oversight and align Japan’s system with international best practices.

## Introduction

**Key Messages**

**Implications for policy makers**

- Replace self-regulatory transparency guidelines with mandatory disclosure laws that require all medical device companies to report financial relationships with healthcare providers through a standardized reporting system.
- Establish a centralized, government-operated public database with machine-readable formats and robust search functionality to enable accountability through effective public monitoring of industry payments.
- Require both healthcare institutions and industry to disclose device costs, selection rationales, and financial relationships in order to reduce information asymmetry and mitigate potential conflicts of interest (COIs).

**Implications for the public**

This study revealed extensive financial relationships between medical device companies and healthcare professionals (HCPs) in Japan, totaling nearly $1 billion over four years. These relationships may influence which medical devices your doctor recommends or your hospital purchases. The research found significant transparency problems, with scattered, inconsistent reporting that makes it difficult for patients, researchers, and regulators to understand these financial ties. Unlike countries with centralized databases of industry payments, Japan relies on a self-regulatory system that lacks standardization and accessibility. By pushing for stronger transparency requirements, patients can gain better insights into potential conflicts of interest (COIs) that might affect their care or result in wasteful public spending. Improving disclosure systems is a first but necessary step in ensuring that medical decisions are based on clinical evidence and patient needs rather than financial relationships between industry and healthcare providers.

Financial relationships between the medical industry and healthcare professionals (HCPs) or healthcare organizations (HCOs) have long attracted attention due to their potential to create conflicts of interest (COIs) that may affect clinical decision-making, research integrity, and perceptions of professional accountability.^1-6^ While pharmaceutical industry payments to HCPs and HCOs have been studied extensively,^1-3^ the medical device sector has received comparatively less attention, despite its rapid global expansion, which was valued at approximately $518.5 billion in 2022 and projected to reach $886.8 billion by 2032.^7^

 In the United States, substantial financial relationships between medical device manufacturers and physicians have been documented, including large aggregate payment amounts and concentration within selected specialties.^7^ In Europe, publicly available disclosures indicate sizeable payments from device companies to HCOs, most commonly in the form of educational and meeting-related support.^8^ Prior research suggests that such financial relationships may influence clinical choices in certain contexts, underscoring the importance of transparency and public oversight.^9^

 By comparison, in 2022, the Japanese medical device market was worth approximately ¥4.19 trillion ($31.6 billion), roughly one-third the size of the pharmaceutical market.^10-12^ The sector is highly fragmented, encompassing more than 100 product classifications and numerous manufacturers represented by the Japan Federation of Medical Devices Associations (JFMDA). Although JFMDA introduced self-regulatory transparency guidelines in 2012, disclosure remains decentralized across individual company websites, with substantial variation in format, accessibility, and completeness. Public discussion of inappropriate financial relationships, often amplified through media coverage of corporate and institutional misconduct, has further highlighted concerns regarding oversight and accountability in this sector.^8^ Key events in the development of regulations and self-governance in the Japanese medical device industry from 1984 to 2012 are summarized in Table S1.

 A recent study by Murayama et al provided an important cross-sectional snapshot of medical device company payments in Japan for the 2022 fiscal year, distinguishing research and non-research transactions.^13^ The present study extends this important analysis by examining payment disclosures from 2019 to 2022, which covers the pre-pandemic, pandemic, and post-pandemic temporal dynamics.

 Specifically, the objectives of this study were threefold: (1) to describe the magnitude and distribution of financial relationships between medical device companies and healthcare stakeholders in Japan over multiple years; (2) to evaluate the accessibility, quality, and availability of disclosure practices under JFMDA’s self-regulatory framework; and (3) to consider policy implications for improving transparency and accountability. A concise overview of Japan’s regulatory milestones in medical device transparency (1984–2012) is provided in Supplementary file 1.

###  Categories and Sub-categories of Disclosed Payments 

 Under the JFMDA transparency guidelines, companies disclose payments across five major categories (A–E) covering research, academic, professional, and promotional activities. Categories A–C require disclosure of individual recipients and payment amounts, whereas categories D and E are reported only in aggregate form. This standardized classification mirrors Japan’s pharmaceutical disclosure framework and enables cross-industry comparison of payment flows. Detailed definitions and sub-categories are summarized in Table 1.

**Table 1:** Definition of Each Disclosure Category of Medical Device Company Payments

**Nature of Payment**	**Definition**	**Beneficiaries**
A. Research and development expenses		HCOs and HCPs
A1. Specific clinical research expenses	Funds for specific clinical research conducted under the Clinical Research Act	
A2. Research expenses according to ethical guidelines	Funds for life science/medical research targeting human subjects	
A3. Non-clinical research expenses	Funds for research other than life science and medical research targeting people (so-called "basic research")	
A4. Clinical trial expenses	Costs for clinical trials conducted under the GCP/GVP/GPSP regulations	
A5. Post-marketing clinical trial expenses	Costs for post-marketing clinical trials conducted under the GCP/GVP/GPSP regulations	
A6. Failure and infection case reporting expenses	Costs for reports of defects/infectious disease cases under the GCP/GVP/GPSP regulations	
A7. Post-marketing surveillance expenses	Costs for post-marketing surveillance activities	
A8. Other expenses	Costs associated with holding meetings related to research and development	
B. Academic research support expenses		HCOs
B1. Scholarship donation (Shogaku-kifu)	Donations to universities and research institutions for research and educational programs	
B2. Donation to academic and other societies	Donations to professional medical societies to support their operations	
B3. General donation	Donations to non-profit organizations and general incorporated associations to support their operations	
B4. Expenses of co-sponsored conferences	Expenses made to host luncheon seminars and symposia in conferences hosted by professional medical societies	
B5. Other expenses	Other expenses related to academic research support	
C. Lecture, manuscript/writing, and consulting/commissioning fees		HCPs
C1. Lecture fee	Compensation for speaking and chairing in company-run educational events	
C2. Manuscript writing fee/supervising fee	Compensation for writing and supervising articles published by companies	
C3. Consulting/commissioning fee	Compensation for helping promote a specific medical product on the market	
D. Information provision-related expenses		HCPs
D1. Expenses for conferences	Expenses to organize conferences and symposia for product promotion, including travel fees, venue, meal, and reception costs	
D2. Expenses for seminars	Expenses to organize study seminars for product promotion	
D3. Expenses for provision of literature and other products	Expenses for articles and documents in medicine/pharmacy, and advertising products (eg, pens and calendars)	
E. Other expenses		HCOs and/or HCPs
E1. Other expenses	Any expenses not included in items B to D, including for food and beverage, gift, and ceremonies	

Abbreviations: HCOs, healthcare organizations; HCPs, healthcare professionals; GCP, Good Clinical Practice; GVP, Good Vigilance Practice; GPSP, Good Post-marketing Study Practice.

###  Comparison with European Disclosures

 In Europe, medical device companies typically categorize payments using two broad disclosure categories, educational events or other educational grants.^8^ These categories partially overlap with Japan’s Academic Research Support Expenses (Category B). For example, Japan’s Scholarship Donations (B1) would likely fall under the other educational grants category and Co-sponsored Conference Expenses (B4) would likely fall under the educational events category. However, the Japanese framework applies more granular classification rules, further dividing research-related payments into specific subcategories such as A4 (Clinical Trials) and A5 (Post-marketing Clinical Trials), which are generally not disclosed on the European medical device payment disclosure database.

 This distinction highlights key structural differences in disclosure architecture between Japan and Europe. Japan’s framework emphasizes detailed categorization of payment purposes but relies on decentralized, company-level disclosures. In contrast, European medical device industry disclosures typically follow standardized industry formats that facilitate cross-company comparison within the sector. While these standardized formats improve internal consistency of reporting, only a limited number of disclosure items are publicly available in Europe. Understanding how these structural differences influence the practical usability of disclosure data is important for informing future transparency reforms.

## Methods

###  Study Scope and Disclosure Framework

 This study employed a predefined, reproducible sampling frame anchored in the JFMDA and its subsidiary, the Japan Medical Devices Manufacturers Association (MD-net).^10^ The MD-net membership list used for this study was obtained and fixed in August 2024, at the start of data collection. MD-net was selected because it provides a clearly identifiable and operationally complete list of major medical device manufacturers subject to the same self-regulatory disclosure framework, comprising 112 member companies across multiple therapeutic areas.

 To improve coverage of large manufacturers with substantial market presence, five non–MD-net companies were additionally included based on prior evidence of active operations in Japan and publicly available disclosures under comparable transparency standards.^14^ These companies were Smith & Nephew, Zimmer Biomet, Carl Zeiss, Kyocera, and Intuitive Surgical. Following this predefined industry framework and the public accessibility of disclosure records, a total of 117 companies were included in the analysis.

 Japan has no national legislation mandating disclosure of payments from medical device companies to HCPs or HCOs. Instead, disclosures are governed by JFMDA/MD-net self-regulatory guidelines, which recommend annual reporting of recipient categories, payment purposes, and monetary amounts, with retention for at least three years. Reporting is fully decentralized, with each company publishing disclosures independently on its own website, and no external enforcement or verification mechanisms exist. Some MD-net members (eg, Santen Pharmaceutical Co., Ltd.) operate mixed pharmaceutical–device businesses; these companies were retained because medical device–related payments are disclosed under the same framework and cannot be meaningfully separated at the disclosure level.

###  Disclosure, Ascertainment, and Inclusion Criteria

 For each year from 2019 to 2022, companies were considered eligible for analysis if payment disclosure files were publicly accessible on company websites at the time of data collection. Data collection was conducted on an ongoing basis, at the time each company’s disclosure data became available. If disclosures were unavailable, removed, or inaccessible during the collection window, the company-year was recorded as non-disclosing for that year.^15^ As a result, 109 companies were included in 2019 and 2020, 114 in 2021, and 117 in 2022.

 Importantly, we did not attempt retrospective reconstruction of disclosures that were no longer publicly available (eg, via archives or secondary reporting), as such reconstruction would not reflect the functional accessibility of information to regulators, researchers, or the public. This approach aligns with the study’s primary objective of evaluating transparency as implemented in practice, rather than estimating latent or undisclosed payment volumes.

 The period of observation was intentionally restricted to 2019–2022 to capture the pre-pandemic baseline (2019), the acute disruption period (2020), and the subsequent recovery phase (2021–2022). This design enables an assessment of how payment patterns and disclosure practices changed across the COVID-19 shock under a stable self-regulatory transparency framework that minimizes heterogeneity in disclosure practices.

###  Data Processing and Analysis

 For eligible company-years, monetary values were extracted at both the total and category/subcategory levels. First, we assessed whether monetary amounts were disclosed for each category and subcategory. Data extraction followed a standardized protocol adapted to the disclosure format: (1) Tabula-assisted extraction with manual verification for PDF reports, and (2) manual standardized entry for decentralized web-based disclosures. Extracted variables included company name, payment category and subcategory, payment year or date, and monetary value. Payments were then aggregated within each company-year to estimate overall financial engagement with HCPs and HCOs, and category composition was calculated as proportions of annual totals. All amounts were converted to U.S. dollars using the 2022 average Telegraphic Transfer Selling exchange rate (132.43 JPY/USD).

 Next, because payment distributions were highly skewed, we summarized data using totals, medians, and interquartile ranges (IQRs) (25th–75th percentiles). Temporal patterns were examined descriptively across the four-year period, using 2019 as a pre-COVID-19 reference point, without causal inference.^16^

 Given the highly right-skewed distribution of payment amounts, payment concentration was additionally summarized using the Gini coefficient as a secondary descriptive statistic, calculated for each year and payment category/sub-category. The Gini coefficient provides a scale-independent measure of distributional concentration that complements summary statistics such as totals, medians, and IQRs.

 Finally, disclosure transparency was evaluated using an adapted version of the framework proposed by Ozieranski et al, originally developed for centralized disclosure systems.^17^ The framework was modified to reflect Japan’s decentralized reporting structure and operationalized across three domains: accessibility, quality, and availability. This assessment was conducted between September and October 2024. All 117 companies were included in this transparency analysis.

 Accessibility assessed the practical ease of locating, searching, and extracting disclosure data; quality evaluated the completeness and clarity of reported information; and availability captured the coverage of recipient groups, payment areas, and multi-year retention of disclosures. The adapted instrument comprised 14 prespecified items scored using binary or ordinal criteria. To avoid over-interpretation of individual checklist items, analyses emphasized domain-level patterns rather than fine-grained item-level comparisons. Coding was conducted by a trained research assistant and independently cross-checked by a second assistant; discrepancies were resolved by Ozaki through consensus.

## Results

Table 2 presents the data disclosure status among companies and across sub-categories from 2019 to 2022, showing an increasing trend in disclosure practices. The number of companies, defined as companies expected to disclose financial interactions due to their participation in Japan’s medical device industry, increased from 109 in 2019 to 117 in 2022. Of these, the number of companies making disclosures increased from 78 (71.6%) to 90 (76.9%), with 73 (62.4%) companies consistently releasing data throughout the four years. The detailed breakdown reveals varying disclosure rates across different sub-categories, with some categories like “Category B: Academic research support expenses,” “Category C: Lecture, manuscript/writing, and consulting/commissioning fees,” and “Category D: Information provision-related expenses” showing consistently high disclosure rates.

**Table 2:** Data Disclosure Status Across Companies by Sub-Category of Payment

**Nature of Payment**	**2019**	**2020**	**2021**	**2022**
**Overall summary**				
Target companies (N)	109	109	114	117
Companies with disclosures (N, %)^a^	78 (71.6%)	82 (75.2%)	86 (75.4%)	90 (76.9%)
**Detailed breakdown by item**^b^				
A. Research and development expenses	68^c^	73	79	84
A1. Specific clinical research expenses (N, %)	38 (55.9%)	46 (63%)	49 (62%)	54 (64.3%)
A2. Research expenses according to ethical guidelines (N, %)	44 (64.7%)	53 (72.6%)	56 (70.9%)	58 (69%)
A3. Non-clinical research expenses (N, %)	43 (63.2%)	54 (74%)	61 (77.2%)	61 (72.6%)
A4. Clinical trial expenses (N, %)	54 (79.4%)	54 (74%)	55 (69.6%)	58 (69%)
A5. Post-marketing clinical trial expenses (N, %)	38 (55.9%)	40 (54.8%)	46 (58.2%)	46 (54.8%)
A6. Failure and infection case reporting expenses (N, %)	44 (64.7%)	46 (63%)	53 (67.1%)	56 (66.7%)
A7. Post-marketing surveillance expenses (N, %)	56 (82.4%)	58 (79.5%)	60 (75.9%)	65 (77.4%)
A8. Other expenses (N, %)	62 (91.2%)	60 (82.2%)	65 (82.3%)	64 (76.2%)
B. Academic research support expenses	71^c^	79	83	88
B1. Scholarship donation (Shogaku-kifu) (N, %)	67 (94.4%)	73 (92.4%)	76 (91.6%)	72 (81.8%)
B2. Donation to academic and other societies (N, %)	68 (95.8%)	70 (88.6%)	73 (88%)	78 (88.6%)
B3. General donation (N, %)	66 (93%)	70 (88.6%)	72 (86.7%)	76 (86.4%)
B4. Expenses of co-sponsored conferences (N, %)	69 (97.2%)	74 (93.7%)	79 (95.2%)	83 (94.3%)
B5. Other expenses (N, %)	-	-	-	1 (1.1%)
C. Lecture, manuscript/writing, and consulting/commissioning fees	70^d^	80	85	88
C1. Lecture fee (N, %)	68 (97.1%)	75 (93.8%)	83 (97.6%)	86 (97.7%)
C2. Manuscript writing fee/supervising fee (N, %)	68 (97.1%)	73 (91.2%)	78 (91.8%)	80 (90.9%)
C3. Consulting/commissioning fee (N, %)	67 (95.7%)	75 (93.8%)	79 (92.9%)	82 (93.2%)
D. Information provision-related expenses	68^d^	77	82	87
D1. Expenses for conferences (N, %)	67 (98.5%)	73 (94.8%)	80 (97.6%)	84 (96.6%)
D2. Expenses for seminars (N, %)	67 (98.5%)	73 (94.8%)	78 (95.1%)	82 (94.3%)
D3. Expenses for provision of literature and other products (N, %)	61 (89.7%)	65 (84.4%)	72 (87.8%)	73 (83.9%)
E. Other expenses				
E1. Other expenses (N, %)	72^d^	80	84	89

^a^ For each year from 2019 to 2022, companies were considered eligible for analysis if payment disclosure files were publicly accessible on company websites at the time of data collection; ^b^ Numbers indicate the number of companies that disclosed specific payment amounts in each category and subcategory for the corresponding year. Percentages were calculated using, as the denominator, the number of companies that disclosed specific monetary amounts for each major category, and, as the numerator, the number of companies that disclosed specific monetary amounts for each subcategory. When specific numerical values were not reported, it was not possible to determine whether the data did not exist or were simply not disclosed; such cases were therefore excluded from the analysis; ^c^ For four companies, data could not be obtained for the category and its directly subordinate subcategories during the disclosure period; ^d^ For five companies, data could not be obtained for the category and its directly subordinate subcategories during the disclosure period.

Table 3 summarizes annual payments by medical device companies in Japan from 2019 to 2022, totaling $942 317 312 across five major categories. Academic research support expenses (Category B) accounted for the largest share at $310 657 526 (33.0%), followed by information provision–related expenses (Category D) at $237 542 031 (25.2%) and research and development expenses (Category A) at $204 821 792 (21.7%). Lecture, manuscript/writing, and consulting/commissioning fees (Category C) comprised $163 125 727 (17.3%), while other expenses (Category E) represented $26 170 236 (2.8%). Within categories, major subcomponents included scholarship donations (B1: $145 809 632; 46.9% of Category B), conference-related expenses (D1: $147 549 569; 62.1% of Category D), and lecture fees (C1: $107 442 233; 65.9% of Category C).

**Table 3:** Annual Medical Device Company Payments by Category and Sub-category in Japan, 2019-2022

**Payment Categories and Sub-categories (USD)**	**2019-2022 Total (%)**^a^	**2019**	**2020 (%)**^b^	**2021 (%)**^b^	**2022 (%)**^b^
**All categories total**	942 317 312	281 892 641	196 785 797 (-30.2%)	214 328 218 (-24.0%)	249 310 656 (-11.6%)
A. Research and development expenses	204 821 792 (21.7%)	48 995 740	53 121 331 (8.4%)	47 614 996 (-2.8%)	55 089 724 (12.4%)
A1. Specific clinical research expenses (N, %)	16 400 845 (8.0%)	4 774 490	4 224 082 (-11.5%)	3 720 943 (-22.1%)	3 681 330 (-22.9%)
A2. Research expenses according to ethical guidelines (N, %)	33 616 411 (16.4%)	6 384 192	8 993 229 (40.9%)	9 646 967 (51.1%)	8 592 024 (34.6%)
A3. Non-clinical research expenses (N, %)	31 924 074 (15.6%)	5 947 259	8 172 577 (37.4%)	7 534 609 (26.7%)	10 269 628 (72.7%)
A4. Clinical trial expenses (N, %)	83 003 532 (40.5%)	20 782 032	23 488 849 (13.0%)	16 375 484 (-21.2%)	22 357 167 (7.6%)
A5. Post-marketing clinical trial expenses (N, %)	2 324 626 (1.1%)	855 082	201 338 (-76.5%)	430 117 (-49.7%)	838 090 (-2.0%)
A6. Failure and infection case reporting expenses (N, %)	152 327 (0.1%)	49 843	34 141 (-31.5%)	34 652 (-30.5%)	33 692 (-32.4%)
A7. Post-marketing surveillance expenses (N, %)	17 477 895 (8.5%)	4 886 764	3 959 073 (-19.0%)	4 556 086 (-6.8%)	4 075 971 (-16.6%)
A8. Other expenses (N, %)	19 922 090 (9.7%)	5 316 078	4 048 042 (-23.9%)	5 316 147 (0.0%)	5 241 823 (-1.4%)
B. Academic research support expenses	310 657 526 (33.0%)	83 315 653	73 908 021 (-11.3%)	75 006 487 (-10.0%)	78 427 364 (-5.9%)
B1. Scholarship donation (Shogaku-kifu) (N, %)	145 809 632 (46.9%)	39 218 770	39 419 574 (0.5%)	35 310 402 (-10.0%)	31 860 887 (-18.8%)
B2. Donation to academic and other societies (N, %)	23 093 349 (7.4%)	8 630 933	5 906 865 (-31.6%)	4 484 238 (-48%)	4 071 313 (-52.8%)
B3. General donation (N, %)	26 089 520 (8.4%)	5 676 049	7 608 576 (34.0%)	6 730 311 (18.6%)	6 074 584 (7.0%)
B4. Expenses of co-sponsored conferences (N, %)	115 666 605 (37.2%)	29 789 901	20 973 007 (-29.6%)	28 481 536 (-4.4%)	36 422 161 (22.3%)
B5. Other expenses (N, %)	27 335 (0.0%)	-	-	-	27 335 (-)
C. Lecture, manuscript/writing, and consulting/commissioning fees	163 125 727 (17.3%)	39 628 623	30 061 877 (-24.1%)	44 839 787 (13.1%)	48 595 440 (22.6%)
C1. Lecture fee (N, %)	107 442 233 (65.9%)	25 996 763	17 099 926 (-34.2%)	30 176 828 (16.1%)	34 168 715 (31.4%)
C2. Manuscript writing fee/supervising fee (N, %)	8 225 906 (5.0%)	1 735 641	1 995 482 (15.0%)	2 402 841 (38.4%)	2 091 941 (20.5%)
C3. Consulting/commissioning fee (N, %)	47 458 414 (29.1%)	11 896 218	10 966 469 (-7.8%)	12 260 095 (3.1%)	12 335 632 (3.7%)
D. Information provision-related expenses	237 542 031 (25.2%)	97 191 029	35 949 168 (-63.0%)	43 719 376 (-55.0%)	60 682 459 (-37.6%)
D1. Expenses for conferences (N, %)	147 549 569 (62.1%)	64 711 394	19 988 297 (-69.1%)	24 797 140 (-61.7%)	38 052 738 (-41.2%)
D2. Expenses for seminars (N, %)	65 933 711 (27.8%)	26 108 520	9 953 109 (-61.9%)	13 801 241 (-47.1%)	16 070 841 (-38.4%)
D3. Expenses for provision of literature and other products (N, %)	24 060 381 (10.1%)	6 371 115	6 009 436 (-5.7%)	5 120 988 (-19.6%)	6 558 843 (2.9%)
E. Other expenses	26 170 236 (2.8%)	12 761 596	3 745 400 (-70.7%)	3 147 571 (-75.3%)	6 515 669 (-48.9%)
E1. Other expenses (N, %)	26 170 236 (100.0%)	12 761 596	3 745 400 (-70.7%)	3 147 571 (-75.3%)	6 515 669 (-48.9%)

^a^ Using the total payment amounts from 2019 to 2022 as the denominator, percentages for each category represent their share of the overall total, while percentages for each subcategory represent the proportion of each item within its corresponding category; ^b^ To describe changes during the COVID-19 period, 2019 was used as the pre-pandemic baseline. Percentages indicate changes relative to 2019 within each category or subcategory.

 Using 2019 as the pre-pandemic baseline, total payments declined from $281 892 641 in 2019 to $196 785 797 in 2020 (−30.2%), partially recovered to $214 328 218 in 2021 (−24.0%), and further increased to $249 310 656 in 2022 (−11.6%). Information provision–related expenses showed the largest decline, decreasing by 63.0% in 2020 and remaining 37.6% below 2019 levels in 2022. In contrast, Category C exceeded pre-pandemic levels in 2022 (+22.6% vs. 2019), while scholarship donations (B1) steadily decreased from $39 218 770 in 2019 to $31 860 887 in 2022 (−18.8%).

 The Figure reveals a highly concentrated pattern of total payments reported by individual medical device companies. The top 10 companies collectively accounted for approximately 58% of the total, with Medtronic plc leading at $97.5 million (10.3% of total payments), followed by Santen Pharmaceutical Co., Ltd. at $69.4 million (7.4%), and Nipro Corporation at $63.1 million (6.7%). Contrastingly, the bottom 50% of companies together paid less than 5% of the total. The highest-paying company’s total payments ($97.5 million) were more than three times that of the 10th-ranked company ($26.4 million). Company-level data is provided in Figure S1 and Table S2.

**Figure:**
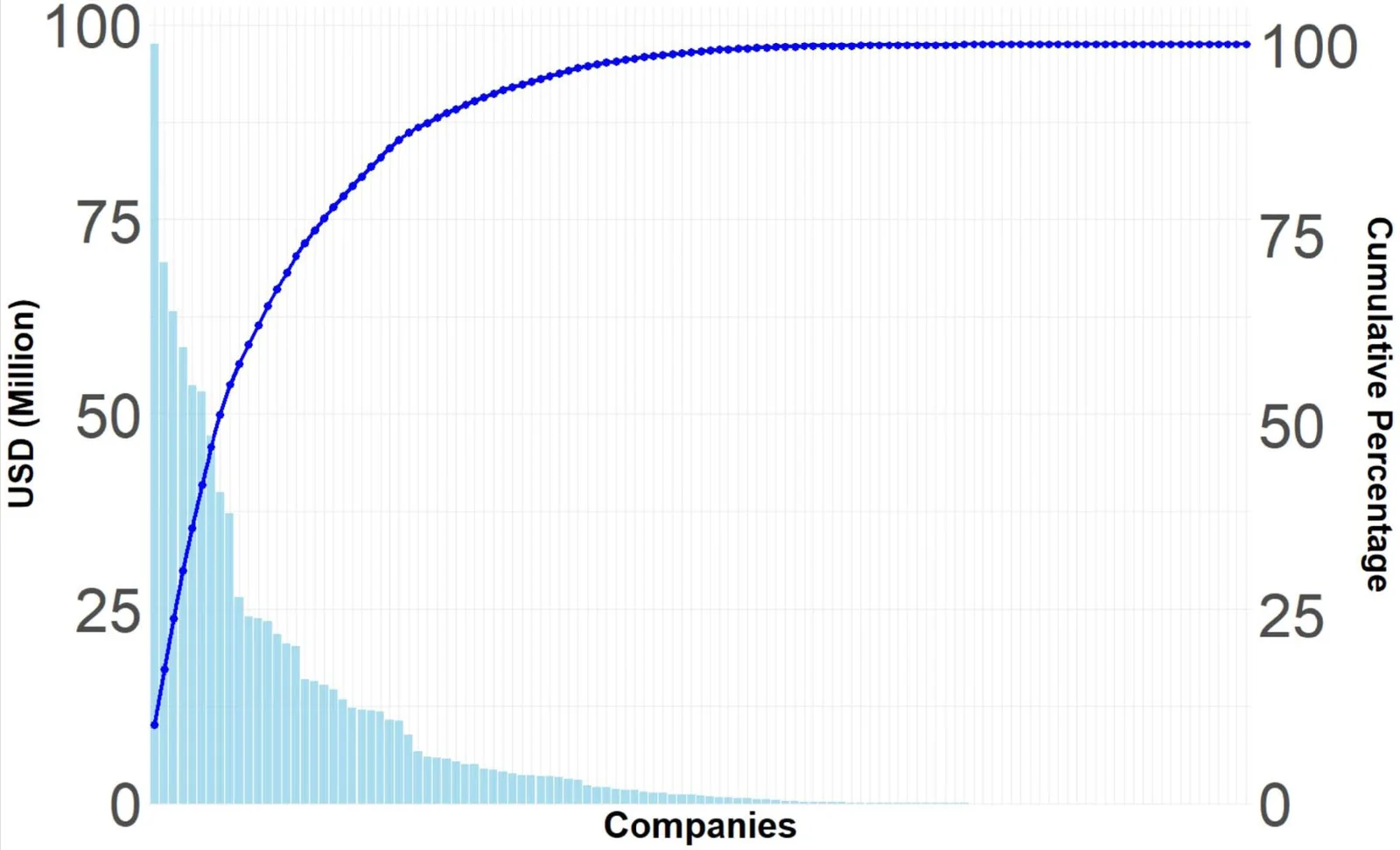
Distribution of Total Medical Device Company Payments by Company in Japan, 2019-2022.

Table 4 presents company-level distributions of payments by major category in Japan from 2019 to 2022, reporting medians, IQRs, and Gini coefficients. Across all categories and years combined, the median payment per company was $92 016 (IQR: $12 772–$486 444), with a high Gini coefficient of 0.79, indicating strong payment concentration. Among categories, academic research support expenses (Category B) had the highest median payment ($257 902; IQR: $46 418–$1 121 662; Gini: 0.71), followed by lecture, manuscript/writing, and consulting/commissioning fees (Category C) ($116 022; IQR: $24 102–$464 168; Gini: 0.74) and information provision–related expenses (Category D) ($105 322; IQR: $16 220–$814 647; Gini: 0.77). Research and development expenses (Category A) showed a lower median ($95 058; IQR: $6781–$553 556) but higher inequality (Gini: 0.80), reflecting concentration among a small number of companies.

**Table 4:** Company-Level Distribution of Payments by Major Category in Japan, 2019–2022 (Median [IQR] and Gini Coefficient)^a^

**Payment Categories and Sub-categories (USD)**	**2019-2022 **	**2019**	**2020**	**2021**	**2022**
All categories (Median, IQR)	92 016 (12 772-486 444)	23 705 (0-180 685)	14 802 (0-82 082)	10 043 (0-91 238)	11 640 (0-98 230)
All categories (Gini coefficient)	0.79	0.83	0.84	0.85	0.85
A. Research and development expenses					
Median (IQR)	95 058 (6781-553 556)	151 (0-68 927)	0 (0-40 144)	0 (0-31 883)	0 (0-38 050)
Gini coefficient	0.80	0.87	0.90	0.90	0.91
B. Academic research support expenses					
Median (IQR)	257 902 (46 418-1 121 662)	48 035 (7797-311 168)	39 891 (3776-233 942)	30 678 (2017-216 328)	25 882 (1521-225 784)
Gini coefficient	0.71	0.78	0.80	0.80	0.81
C. Lecture, manuscript/writing, and consulting/commissioning fees					
Median (IQR)	116 022 (24 102-464 168)	31 216 (6046-169 580)	25 727 (4639-82 381)	23 943 (3512-111 506)	23 497 (3298-111 502)
Gini coefficient	0.74	0.79	0.79	0.82	0.83
D. Information provision-related expenses					
Median (IQR)	105 322 (16 220-814 647)	50 230 (3101-350 209)	18 642 (1280-123 759)	13 567 (323-88 446)	22 922 (834-115 491)
Gini coefficient	0.77	0.80	0.81	0.84	0.83
E. Other expenses					
Median (IQR)	19 663 (3644-82 770)	60 160 (14 015-215 362)	12 772 (3277-61 213)	8360 (1736-45 829)	20 160 (3550-80 762)
Gini coefficient	0.74	0.67	0.70	0.69	0.72

Abbreviation: IQR, interquartile range. ^a^ Median and IQR was calculated across all companies in the analytic sample for each category and year (including companies with zero payments). Gini coefficients quantify payment concentration across companies within each year and category.

 Over time, median total payments declined from $23 705 (IQR: $0–$18 685; Gini: 0.83) in 2019 to $14 802 (IQR: $0–$82 082; Gini: 0.84) in 2020 and $10 043 (IQR: $0–$91 238; Gini: 0.85) in 2021, before partially recovering to $11 640 (IQR: $0–$98 230; Gini: 0.85) in 2022. Despite these fluctuations, Gini coefficients remained consistently high (0.83–0.85), indicating persistent concentration of payments across companies throughout the study period.

 Table S3 extends the company-level analysis by disaggregating each major payment category into its constituent subcategories. Overall, the distributional patterns observed at the major category level were largely preserved at the subcategory level. In subcategories that dominated their parent categories, payment concentration was particularly pronounced, reinforcing the tendency for a small subset of companies to account for a disproportionate share of payments.

Table 5 summarizes the accessibility, quality, and availability of payment disclosures by medical device companies in Japan. Among the 117 companies assessed, 92 (78.6%) disclosed payment information in at least one year during the 2019–2022 study period. This figure does not necessarily correspond to the number of companies disclosing data in 2022, as data availability varied at the time of assessment (September–October 2024). For example, some companies that had not disclosed data in earlier years began disclosing payment information for 2023, while others did not consistently disclose data across all study years. Most disclosures were provided as PDFs, with 46 companies (50.0%) using machine-readable PDFs and 16 (17.4%) using image-based PDFs, while only 30 companies (32.6%) used more accessible formats such as webpages, XLS, or CSV files. Although all disclosing companies (92, 100.0%) provided online methodology notes and clear definitions of relevant terms, none adopted a standardized disclosure template, enabled database download, or offered customizable summary statistics.

**Table 5:** Assessment of Accessibility, Quality, and Availability of Medical Device Company Payment Disclosures in Japan

**Variables**	**No. of Companies (N = 117)**
**Accessibility**	
Database format (N, %)	92 (78.6%)
Webpage, XLS or CSV	30 (32.6%)
Machine Readable PDFs	46 (50.0%)
Image-based PDFs	16 (17.4%)
Database structure: Does the data from all companies follow a single template consistently? (N, %)	92 (78.6%)
Yes	0 (0.0%)
A format provided by Dentsu Corporation	14 (15.2%)
Anything else	78 (84.8%)
Database searchability (N, %)	92 (78.6%)
Advanced search available	0 (0.0%)
Limited search available	24 (26.1%)
No search available	68 (73.9%)
Customizable summary statistics: Does the database offer users the possibility of generating real-time, dynamic data summaries based on selected database characteristics? (N, %)	92 (78.6%)
Yes	0 (0.0%)
N/A	0 (0.0%)
No	92 (100.0%)
Unique identifiers (ID) (N, %)	92 (78.6%)
All donors and recipients	0 (0.0%)
Some donors or recipients	0 (0.0%)
No unique identifiers	92 (100.0%)
Downloadability: Can the database be downloaded (eg, as a single CSV or XLS file) for further analysis? (N, %)	92 (78.6%)
Yes	0 (0.0%)
N/A	0 (0.0%)
No	92 (100.0%)
Methodology notes (N, %)	92 (78.6%)
Available online	92 (100.0%)
Available on request	0 (0.0%)
Not available	0 (0.0%)
Year limit: Are disclosures removed after a certain number of years? (N, %)	92 (78.6%)
No	55 (59.8%)
N/A	7 (7.6%)
Yes	30 (32.6%)
Definitions (N, %)	92 (78.6%)
Clear and comprehensive definitions are provided for all relevant terminology (eg, HCO, payment categories)	92 (100.0%)
Definitions are provided for some relevant terminology	0 (0.0%)
No definitions are provided for relevant terminology	0 (0.0%)
**Quality**	
Spectrum of disclosed characteristics (N, %)	92 (78.6%)
All characteristics from the US Open Payments database covered	0 (0.0%)
Some of the characteristics from the US Open Payments database covered	92 (100.0%)
None of the characteristics from the US Open Payments database covered	0 (0.0%)
Aggregation of payments (N, %)	92 (78.6%)
All payments itemized	0 (0.0%)
Some payments itemized, others aggregated	86 (93.5%)
All payments aggregated	6 (6.5%)
Inclusion of taxes (N, %)	92 (78.6%)
Single rule for all companies and payments	0 (0.0%)
No single rule, each company sets its own rules for VAT reporting which are published separately from payment disclosures	40 (43.5%)
Rules around tax reporting are unclear	52 (56.5%)
**Availability**	
Breadth of recipients (N, %)	92 (78.6%)
All of the following groups of recipients are included: Patient organizations, HCOs, HCPs	5 (5.4%)
N/A	0 (0.0%)
Some of the following groups of recipients are not included: Patient organizations, HCOs, HCPs	87 (94.6%)
Breadth of payment areas (N, %)	92 (78.6%)
All of the following types of payments included: education, research, consulting, gifts, charitable donations, ownership and investment interest	0 (0.0%)
N/A	0 (0.0%)
Some of the following types of payments are not included: education, research, consulting, gifts, charitable donations, ownership and investment interest	92 (100.0%)

Abbreviations: HCOs, healthcare organizations; HCPs, healthcare professionals; VAT, value-added tax.

 Searchability was limited: 68 companies (73.9%) provided no search function, and 24 (26.1%) offered only limited search capabilities. Unique identifiers for donors and recipients were largely absent, with all companies (92, 100.0%) lacking such identifiers. Regarding data retention, 55 companies (59.8%) did not specify a time limit for disclosure availability, while 30 (32.6%) removed disclosures after a certain period.

 In terms of quality, most companies (86, 93.5%) disclosed a mix of itemized and aggregated payments. Tax reporting practices were inconsistent, with unclear rules in 52 companies (56.5%). Availability was also limited: only 5 companies (5.4%) disclosed payments to all relevant recipient groups, and none disclosed all payment types across education, research, consulting, gifts, charitable donations, and ownership or investment interests, indicating substantial gaps in the scope and usability of disclosures.

## Discussion

###  Summary of Payment Patterns and Comparison With Japan’s Pharmaceutical Industry 

 This study extends prior single-year descriptions of medical device industry payments in Japan by examining multi-year dynamics from 2019 to 2022, thereby capturing changes before, during, and after the COVID-19 pandemic.^13^

 Across the four-year period, total payments amounted to $942.3 million (¥124.8 billion), indicating a non-negligible level of financial interaction between medical device companies and healthcare stakeholders. However, this scale remains substantially smaller than that of the pharmaceutical sector, where industry payments exceeded $1.7 billion in a single year.^16^ This contrast underscores important sectoral differences and cautions against interpreting absolute payment volumes in isolation from their wider context.

 Academic research support expenses (Category B) constituted the largest share of payments (33.0%), driven primarily by co-sponsored events (B4), which increased from $29.8 million in 2019 to $36.4 million in 2022. In contrast, scholarship donations (B1) declined by 18.8% over the same period. Similar declines have been reported in the pharmaceutical sector following heightened regulatory scrutiny and legal actions addressing inappropriate use of scholarship donations.^17-19^ These parallel trends suggest that external oversight, rather than voluntary disclosure alone, plays a central role in shaping industry payment behavior.

 Payment categories appear to have responded heterogeneously to the COVID-19 pandemic. Consulting, lecturing, and manuscript-related fees (Category C) declined initially but exceeded pre-pandemic levels by 2022, whereas information provision–related expenses (Category D), such as conference and hospitality costs, remained 37.6% below 2019 levels. This divergence is consistent with a structural shift toward remote professional engagement and increasing reliance on specialized clinical expertise, particularly for technologically complex devices.^20^ Such category-specific trajectories could be identified only through further analyses spanning multiple years.

 Research and development expenses (Category A) accounted for 21.7% of total payments. While research-related funding is essential for device innovation, the lack of detailed contextual information in current disclosures limits the ability to distinguish independent research support from activities with potential promotional intent, as illustrated by prior regulatory cases involving medical device companies.^8^

 Importantly, payments were highly concentrated: the top 10 companies accounted for approximately 58% of total payments, indicating a structurally skewed distribution dominated by a small number of large manufacturers. Similar concentration patterns have also been reported in analyses of pharmaceutical industry payments to HCPs in Japan, where a small number of companies account for a disproportionate share of total payments.^21^

 Together, these findings demonstrate that Japan’s medical device payment landscape is characterized not only by the magnitude of financial relationships but also by persistent concentration and differential category responses to external shocks. We interpret the observed changes as being consistent with reduced opportunities for in-person activities during the COVID-19 period.

###  Comparison of Payment Patterns and Transparency in the Medical Device Sector in Japan and Europe

 Both Japan and most European countries rely largely on industry self-regulation to govern disclosures of payments from medical device companies to HCPs and HCOs.^15,19,21,22^ However, the two systems differ substantially in their disclosure architecture, particularly with respect to data structure, accessibility, and analytical usability.

 Japan’s governance framework requires companies to report payments using relatively detailed predefined subcategories, including clinical trial expenses (A4) and post-marketing clinical trial expenses (A5). This structure allows relatively precise differentiation of payment purposes, particularly within research and development activities. In contrast, the primary disclosure system in the European medical device sector generally classifies payments using broader reporting categories,^8^ which limits the level of detail available regarding the specific nature of financial relationships. In addition, many research and other types of payments are not reported in the European system, and many European medical device companies do not publicly disclose payments.^8^

 At the same time, the European medical device disclosure framework provides a degree of centralized access through an industry-level database that aggregates company disclosures, enabling cross-company and cross-country analysis of payment patterns.^8^ By comparison, Japan’s disclosure system remains fully decentralized, with payment information dispersed across individual company websites and frequently presented in non-standardized formats. Consequently, although Japan provides greater categorical detail, the decentralized structure substantially reduces accessibility and complicates data aggregation and systematic oversight.

 Our transparency assessment illustrates this trade-off. While Japan offers finer categorical granularity, 94.6% (87 of 92) of companies exhibited incomplete recipient group coverage, and none satisfied international standards for comprehensive itemized disclosure across accessibility, quality, and availability domains. These limitations restrict the practical usability of disclosure data and hinder systematic monitoring of financial relationships.

 Overall, then, Japan’s system prioritizes detailed classification, whereas the European medical device disclosure system emphasizes centralized access but offers more limited detail. From a governance perspective, both approaches remain incomplete: granular data that cannot be easily accessed and standardized data lacking sufficient detail are both limited as transparency mechanisms. Future policy design should therefore integrate both dimensions to improve the functional value of disclosure systems.^17^ Importantly, these limitations do not negate the value of legitimate industry–clinician collaboration, but rather highlight the need for disclosure systems that allow such collaboration to be accountable to the public.

 That said, in both jurisdictions disclosure systems remain weaker than statutory transparency frameworks such as the U.S. Sunshine Act or France’s Loi Bertrand, which mandate standardized and centralized reporting of payments related to medical devices.^17^

###  Policy Implications and Limitations of the Current Self-regulatory Framework 

 As medical device technologies become more complex and increasingly reliant on clinical expertise, financial relationships, such as royalties or licensing arrangements linked to device innovation, between manufacturers and healthcare providers are likely to intensify.^8,23,24^

 Our findings indicate that payments are highly concentrated among few companies and recipients, particularly in specialized fields with limited pools of experts. Such concentration may amplify organizational dependency risks, as previously demonstrated in analyses of financial reliance within healthcare systems.^25^ In these contexts, transparency is a basic stepping stone that can facilitate other policy mechanisms to ensure that financial relationships do not compromise professional judgement, research integrity or communication of treatment benefits to the wider public.

 After more than a decade of self-regulatory disclosure, persistent gaps in accessibility, completeness, and standardization suggest that self-regulation alone is insufficient.^25^ For companies whose products are reimbursed through Japan’s national health insurance system, legally mandated public disclosure, similar to frameworks implemented in the United States and several European countries, should be seriously considered.^14,27^ A centralized, government-run database specifying the purpose, category, and context of payments would substantially enhance transparency and oversight. Such a system should be coupled with systematic monitoring, data quality checks, and meaningful, enforceable penalties for non-compliance.^27,28^

 In parallel, greater transparency regarding medical device performance and pricing is needed to balance promotional influences within clinical settings. Without accessible information on both financial relationships and device-related outcomes, healthcare institutions and the public remain limited in their ability to critically evaluate the role of industry interactions in clinical decision-making.

###  Summary of Policy Implications and Regulatory Recommendations

 Taken altogether, our findings have important implications for health policy and governance in Japan’s medical device sector. First, the scale and persistent concentration of payments, accompanied by substantial limitations in accessibility and standardization, indicate that the current self-regulatory disclosure framework is insufficient to support meaningful public oversight.^28^ Transitioning from industry self-regulation to legally mandated disclosure requirements would likely provide a stronger foundation for transparency and public accountability, particularly for companies whose products are reimbursed through the national health insurance system. Second, the highly decentralized and heterogeneous disclosure practices identified in this study highlight the need for a centrally administered, legally mandated disclosure system overseen by a governmental or independent regulatory body.^28^ Such a system should enable machine-readable access, cross-company comparability, and longitudinal monitoring of financial relationships.^23^ Finally, transparency reforms should be accompanied by routine monitoring and enforceable compliance mechanisms, since disclosure without verification risks becoming tokenistic rather than an effective governance tool.^29,30^

 Collaboration between industry and clinicians can play a legitimate and beneficial role, particularly in device development and post-marketing safety improvement, but only if accompanied by strong transparency governance. However, even comprehensive and appropriately enforced transparency is insufficient to fully protect patient health and research and professional integrity. It is therefore important to use improved disclosed payment data to assess and develop robust, evidence-based policy tools to manage the risks of bias that may arise from industry collaborations.^26^

###  Limitations

 This study has several limitations. First, the analysis relied on payment data self-disclosed by medical device companies, which may be incomplete or selectively reported, potentially leading to an underestimation of the true scale and nature of financial relationships.^26^ Second, for some companies in 2019, incomplete data extraction occurred, which may have further limited the comprehensiveness of the analysis. Third, substantial heterogeneity in disclosure formats and reporting practices limited cross-company comparability and constrained industry-wide analyses. Fourth, in the absence of legally mandated disclosure requirements, as implemented in the United States and some European countries, such as France and Denmark,^23^ it was not possible to externally verify or enforce the accuracy of reported information.

 In addition, the lack of a standardized disclosure framework hindered the assessment of data completeness and internal consistency. Some MD-net member companies also engage in pharmaceutical production (eg, Santen Pharmaceutical Co., Ltd.) while manufacturing medical devices; these companies were retained to preserve coverage of the medical device sector as defined by the disclosure guidelines. Because disclosures are reported at the company level and are not disaggregated by product line or clinical specialty, we could not distinguish payments related to medical versus dental device activities for manufacturers with mixed portfolios. Importantly, the reported figures likely represent a lower bound of industry–healthcare financial interactions, as not all payments may have been disclosed or retained online during the study period.^26^ Moreover, observed changes in disclosure rates over time may partly reflect the removal or archiving of previously disclosed webpages, rather than true changes in disclosure behavior. All analyses in this study were conducted at the company and category levels; therefore, no individual-level conclusions or inferences about the appropriateness of specific payments were made.

 These limitations preclude definitive conclusions regarding the full extent or intent of financial relationships within Japan’s medical device industry. Future research would benefit from standardized, centralized, and legally enforced disclosure systems that enable more accurate, comparable, and verifiable analyses.

## Conclusion

 This study provides a multi-year assessment of financial relationships between Japan’s medical device industry and healthcare stakeholders from 2019 to 2022 ($942.3 million (¥124.8 billion)), to evaluate persistent challenges in transparency and oversight. Payments were highly concentrated among a small number of companies, and disclosure practices remained fragmented and difficult to access, limiting public usability and cross-company comparison.

 Despite detailed categorical reporting, Japan’s decentralized disclosure system lacks standardized formats and comprehensive accessibility, in contrast to centralized databases used in other jurisdictions. These structural limitations constrain the effectiveness of transparency as a governance tool. Addressing these gaps—through standardized reporting and stronger institutional oversight—will be essential for improving accountability while preserving legitimate collaboration between industry and HCPs in support of innovation.

## Acknowledgements

 Masahiro Kami, MD, PhD (Medical Governance Research Institute), provided his constructive opinion of the manuscript; he was not compensated for his work.

## Disclosure of artificial intelligence (AI) use

 We utilized ChatGPT and Claude AI language models solely for English language editing and proofreading. All research design, data analysis, interpretation, and scientific conclusions represent the authors’ original work, for which we maintain full responsibility.

## Ethical issues

 This study was approved by the Ethics Committee of the Medical Governance Research Institute (approval number: MG2024-03). The research utilizes publicly available information disclosed by pharmaceutical and medical device companies regarding their financial relationships with HCPs. Given the nature of the study using publicly available data, and in accordance with the ethical considerations outlined in the research protocol, individual informed consent was waived. The study was conducted in compliance with the Ethical Guidelines for Medical and Health Research Involving Human Subjects in Japan. All data management procedures followed strict security protocols to ensure appropriate handling of information.

## Conflicts of interest

 Akihiko Ozaki received personal fees from Medical Network Systems (MNES), Kyowa Kirin Inc., Becton, Dickinson and Company, Pfizer, Daiichi Sankyo Inc. and Taiho Pharmaceutical Co., Ltd., outside the submitted work. Hiroaki Saito reported receiving personal fees from Taiho Pharmaceutical Co., Ltd., and Tetsuya Tanimoto reported receiving personal fees from Medical Network Systems, outside of the submitted work. Piotr Ozieranski received reimbursement of travel and accommodation expenses from Code Clarity to serve as a panel member at the Code Clarity Annual conference in 2025. No honorarium was received.

## Supplementary files


Supplementary file 1 contains Tables S1-S3 and Figure S1.
