# Supplementary material for: Medical Device Industry Payments to Healthcare Professionals and Organizations in Japan: An Evaluation of Scale, Distribution, and Transparency Practices, 2019-2022
Source: Int J Health Policy Manag. 2026 Jun 1;15:9079. doi: 10.34172/ijhpm.9079 (PMC13729721; doi:10.34172/ijhpm.9079)
Supplement: Supplementary file 1 [file ijhpm-15-9079-s001.pdf]

Article title: Medical Device Industry Payments to Healthcare Professionals and Organizations in Japan: An Evaluation of Scale, Distribution, and Transparency Practices, 2019-2022

Journal name: International Journal of Health Policy and Management (IJHPM)

Authors' information: Akemi Hara[1]¶, Sunil Shrestha[2]¶, Hiroaki Saito[1,3], James Larkin[4], Frank Moriarty[4], Piotr Ozieranski[5], Rajeev Shrestha[6], Tetsuya Tanimoto[1,7], Akihiko Ozaki[1,8]*

[1]Medical Governance Research Institute, Tokyo, Japan.

[2]Department of Research and Academics, Kathmandu Cancer Center, Bhaktapur, Nepal.

[3]Department of Internal Medicine, Soma Central Hospital, Fukushima, Japan.

[4]Department of General Practice, Royal College of Surgeons in Ireland, University of Medicine and Health Sciences, Dublin, Ireland.

[5]Department of Social and Policy Sciences, University of Bath, Bath, UK.

[6]NIHR Newcastle Patient Safety Research Collaboration, Newcastle University, Newcastle upon Tyne, UK.

[7]Navitas Clinic Kawasaki, Kanagawa, Japan.

[8]Breast and Thyroid Center, Jyoban Hospital of Tokiwa Foundation, Iwaki, Japan.

***Correspondence to:** Akihiko Ozaki; Email: ozakiakihiko@gmail.com

¶Both authors contributed equally to this paper.

Citation: Hara A, Shrestha S, Saito H, et al. Medical device industry payments to healthcare professionals and organizations in Japan: an evaluation of scale, distribution, and transparency practices, 2019-2022. Int J Health Policy Manag. 2026;15:9079. doi:10.34172/ijhpm.9079

Supplementary file 1

Table S1. Key events in the development of medical device industry regulations and self-governance in Japan, 1984-2012

Year	Event
1984	JFMDA was established
1984	Fair Trade Commission establishes industry-specific premium notification for prescription pharmaceuticals
1991	A bribery scandal involving X-ray CT scanners and cardiac pacemakers involving the national university hospitals
1992	Ministry of Health issues administrative guidance requiring the medical device industry to improve business practices
1993	JFMDA establishes 'Code of Ethics'
1997	JFMDA establishes 'Medical Device Industry Promotion Code'
1998	Three significant developments:<br>- Fair Trade Commission extends regulations to include medical devices in 'Restrictions on Premium Offerings in Prescription Pharmaceutical, Medical Device, and Clinical Laboratory Industries'<br>- 'Fair Competition Code Regarding Restrictions on Premium Offerings in Medical Device Industry' is established<br>- Medical Devices Fair Trade Council is founded
1999	Fair Competition Code implemented
2005	Related ministries and medical societies begin developing conflict-of-interest management guidelines
2007	JFMDA establishes 'Corporate Charter.'
2011	Japan Pharmaceutical Manufacturers Association establishes 'Transparency Guidelines for the Relationship between Corporate Activities and Medical Institutions'
2012	JFMDA establishes 'Transparency Guidelines for the Relationship between the Medical Device Industry and Medical Institutions'

JFMDA= Japan Federation of Medical Devices Associations

Table S2. The total amount of medical device expenditures for each company from 2019 to 2022

Company name	A (million $)	B (million $)	C (million $)	D (million $)	E (million $)	Total (million $)	Cumulative Proportion
Medtronic Japan Co., Ltd.	12	39.3	21.8	22.2	2.1	97.5	10.3
Santen Pharmaceutical Co., Ltd.	32.4	14.5	5.2	17	0.3	69.4	17.7
Nipro Corporation	32.2	20.3	5.9	4.1	0.7	63.1	24.4
Abbott Medical Japan LLC	5.8	27	13	10.5	2.4	58.6	30.6
Johnson & Johnson K.K.	1.8	16.4	13.1	20	2.4	53.7	36.3
Terumo Corporation	5.7	18.7	15.5	11.9	1	52.8	41.9
KYORIN Pharmaceutical Co., Ltd.	6.9	5.5	7.9	26.2	0.8	47.3	47
Boston Scientific Japan K.K.	8.2	15.6	7	7.7	1.5	39.9	51.2
KAKEN PHARMACEUTICAL CO., LTD.	13.8	5.9	5.7	11.7	0.1	37.2	55.1
Olympus Corporation	6.9	9.4	4.8	5.1	0.2	26.4	58
Sysmex Corporation	13.1	5.3	1.1	4.1	0.5	24	60.5
Stryker Japan K.K.	1.8	11.1	3.4	6.7	0.8	23.8	63
Otsuka Pharmaceutical Factory, Inc.	4.3	4.1	4	10.5	0.5	23.4	65.5
Edwards Lifesciences Japan LLC	2.8	7.1	4.8	6.1	1	21.8	67.8
Sanwa Kagaku Kenkyusho Co., Ltd.	9.5	3.1	2.5	5.2	0.2	20.5	70
Zimmer Biomet G.K.	0.5	9.8	2.6	6.2	1	20.1	72.1
Kyocera Corporation	4.8	9.9	0.4	0.3	0.6	16	73.8
Kracie Pharmaceutical, Ltd.	1.9	1.4	1.4	10.5	0.5	15.7	75.5
Philips Japan, Ltd.	1.6	8.6	1.8	2.6	0.6	15.2	77.1
Kaneka Medix Corporation	0.1	4.5	7.2	2	0.9	14.7	78.7
Allergan Japan K.K.	6.7	0.4	0.9	5	0.2	13.3	80.1
Alcon Japan Ltd.	3.4	3.5	1.5	3.6	0.3	12.3	81.4
HOYA Corporation	2.6	4.8	2.3	1.4	1	12.1	82.7
Fukuda Denshi Co., Ltd.	2.2	6	2.3	0.9	0.6	11.9	83.9
Baxter Limited	1.2	5.5	2	2.6	0.6	11.8	85.2
Maruishi Pharmaceutical Co., Ltd.	2.8	1.1	0.7	6.1	0	10.8	86.3
Abbott Japan Co., Ltd.	0.8	2.9	2	4.8	0.3	10.7	87.5
Smith & Nephew K.K.	0.6	4.4	1.2	2.4	0.2	8.8	88.4
Nihon Kohden Corporation	0.4	2.9	1.5	1.4	0.3	6.6	89.1
Intuitive Surgical G.K.	0.3	2.5	2.1	1.1	0	6	89.7

AMO Japan K.K.	0.5	2.7	0.6	1.9	0.3	5.9	90.4
Century Medical, Inc.	0.3	3.3	1.3	0.8	0.1	5.8	91
W. L. Gore & Associates Japan K.K.	0.6	3	1	0.7	0.1	5.4	91.5
Alfresa Pharma Corporation	1.9	0.7	0.7	1.8	0	5.1	92.1
NIDEK CO., LTD.	1.3	3.2	0.2	0.2	0.1	5	92.6
Cook Japan K.K.	0.1	1	2	1.1	0.3	4.5	93.1
Senko Medical Instrument Mfg. Co., Ltd.	0.8	3	0.4	0.1	0	4.3	93.6
Asahi Kasei Medical Co., Ltd.	0.9	2	0.4	0.4	0.2	4	94
JMS Co., Ltd.	0.2	1.9	1.1	0.4	0.2	3.9	94.4
Nitoms, Inc.	3.2	0.2	0.1	0	0	3.6	94.8
Medicon Inc.	0.3	1.1	1	1	0.2	3.6	95.1
ALCARE Co., Ltd.	0.3	1	0.4	1.8	0	3.5	95.5
Siemens Healthcare Diagnostics K.K.	0.3	2.3	0.2	0.2	0.5	3.5	95.9
SB-KAWASUMI LABORATORIES, INC.	0.9	0.7	1.4	0.3	0.1	3.4	96.2
Nikkiso Co., Ltd.	0.2	2	0.2	0.2	0.4	3.1	96.6
B. Braun Aesculap Japan K.K.	0.4	1.5	0.4	0.5	0.2	3	96.9
OrbusNeich Medical K.K.	0.1	0.3	0.3	1.2	0.3	2.3	97.1
MC Healthcare Group Co., Ltd.	0.3	0.9	0.5	0.4	0.1	2.1	97.4
Becton, Dickinson and Company Japan	0.6	0.7	0.6	0.1	0	2.1	97.6
LifeScan Japan K.K.	0	0.9	0.3	0.6	0.1	1.8	97.8
Toray Medical Co., Ltd.	0.2	0.5	0.5	0.5	0.1	1.8	98
Hogy Medical Co., Ltd.	0.6	0.5	0.1	0.4	0.1	1.7	98.2
Convatec Japan K.K.	0	0.4	0.3	0.3	0.4	1.5	98.3
LivaNova Japan K.K.	0.2	0.4	0.6	0.2	0	1.4	98.5
Integra Japan Co., Ltd.	0	0.4	0.4	0.5	0	1.4	98.6
Beckman Coulter K.K.	0.1	0.4	0.3	0.4	0	1.1	98.7
ATOM MEDICAL CORPORATION	0.4	0.4	0.1	0.1	0.2	1.1	98.9
Create Medic Co., Ltd.	1	0	0	0	0	1.1	99
KCI Japan G.K.	0	0.5	0.2	0.3	0	1	99.1
Getinge Japan K.K.	0.1	0.5	0.2	0.1	0.1	0.9	99.2
Cardinal Health Japan G.K.	0.1	0.3	0.3	0.2	0	0.9	99.3
Saraya Co., Ltd.	0.7	0	0.1	0	0	0.8	99.4
Sumitomo Bakelite Co., Ltd.	0.2	0.2	0.2	0.1	0	0.7	99.4
Gunze Medical Limited	0	0.4	0.1	0.1	0	0.7	99.5
Daiken Medical Co., Ltd.	0.3	0.1	0.1	0	0.1	0.6	99.6

Sakura Seiki Co., Ltd.	0	0.4	0	0.1	0	0.5	99.6
3M Japan Limited	0	0.2	0.2	0	0	0.5	99.7
Glaukos Japan G.K.	0.1	0.1	0.1	0	0	0.4	99.7
Smiths Medical Japan Ltd.	0	0.2	0	0.1	0	0.3	99.7
Corcym Japan K.K.	0	0	0.2	0	0	0.3	99.8
embecta G.K.	0	0	0.1	0	0	0.2	99.8
Avanos Medical Japan Inc.	0	0.1	0.1	0	0	0.2	99.8
Teleflex Medical Japan G.K.	0	0.1	0	0	0	0.2	99.8
Fuji Systems Corporation	0	0	0.1	0	0	0.2	99.8
Bear Medic Corporation	0	0.1	0	0	0.1	0.2	99.9
Merit Medical Japan Co., Ltd.	0	0.1	0	0	0	0.2	99.9
SIGMAX Co., Ltd.	0	0.1	0	0	0	0.2	99.9
O&M Halyard Japan G.K.	0	0.1	0	0	0	0.1	99.9
aglis corporation	0	0	0	0	0.1	0.1	99.9
LiveDo Corporation	0	0.1	0	0	0	0.1	99.9
Dräger Japan Ltd.	0	0	0	0	0	0.1	99.9
Kawamoto Corporation	0	NA	0.1	NA	0	0.1	100
Hollister Japan K.K.	0	0	0	0	0	0.1	100
TOP Corporation	0	0	0	0	0.1	0.1	100
Osaki Medical Corporation	NA	0	0	NA	0	0.1	100
ASP Japan G.K.	NA	0	0	0	0	0.1	100
Radiometer K.K.	0	0	0	0	0	0.1	100
David Medical Instrument Co., Ltd.	0	0	0	0	0	0	100
Unisys Corporation Japan G.K.	0	0	0	0	0	0	100
Medline Japan G.K.	NA	0	NA	0	0	0	100
Hakujuji Co., Ltd.	NA	0	0	0	NA	0	100
Ansell Healthcare Japan Co., Ltd.	0	0	0	0	0	0	100
Japan Medical Products Co., Ltd.	0	0	0	0	0	0	100
Nissho Sangyo Co., Ltd.	0	0	0	0	0	0	100
ARKRAY Marketing Inc.	NA	NA	NA	NA	NA	NA	NA
AS ONE Corporation	NA	NA	NA	NA	NA	NA	NA
AMCO INC.	NA	NA	NA	NA	NA	NA	NA
Iwatsuki Co., Ltd.	NA	NA	NA	NA	NA	NA	NA
Kao Professional Services Co., Ltd.	NA	NA	NA	NA	NA	NA	NA
Takenaka Medical Co., Ltd.	NA	NA	NA	NA	NA	NA	NA

Nemoto Kyorindo Co., Ltd.	NA	NA	NA	NA	NA	NA	NA
Bayer Medical Care Ltd.	NA	NA	NA	NA	NA	NA	NA
Hakujuji Hanbai Co., Ltd.	NA	NA	NA	NA	NA	NA	NA
Hitachi High-Tech Fielding Corporation	NA	NA	NA	NA	NA	NA	NA
Bethel Plus Co., Ltd.	NA	NA	NA	NA	NA	NA	NA
Matsuyoshi & Co., Ltd.	NA	NA	NA	NA	NA	NA	NA
Metz Co., Ltd.	NA	NA	NA	NA	NA	NA	NA
Medicom Japan Co., Ltd.	NA	NA	NA	NA	NA	NA	NA
Metran Co., Ltd.	NA	NA	NA	NA	NA	NA	NA
Moraine Corporation	NA	NA	NA	NA	NA	NA	NA
Yoshida Pharmaceutical Company Limited	NA	NA	NA	NA	NA	NA	NA
Utsunomiya Seisaku Co., Ltd.	NA	NA	NA	NA	NA	NA	NA
MTG Co., Ltd.	NA	NA	NA	NA	NA	NA	NA
Ambu K.K.	NA	NA	NA	NA	NA	NA	NA
Shockwave Medical Japan K.K.	NA	NA	NA	NA	NA	NA	NA
Coloplast K.K.	NA	NA	NA	NA	NA	NA	NA
NUTRI Co., Ltd.	NA	NA	NA	NA	NA	NA	NA

Table S3. Distribution of company-level payment amounts by subcategory for the entire study period and by year

Payment categories and sub-categories (USD)	2019-2022	2019	2020	2021	2022
**A. Research and development expenses**					
A1. Specific clinical research expenses (Median, IQR)	0 (0 - 62599)	0 (0 - 48241)	1888 (0 - 47197)	0 (0 - 81684)	0 (0 - 63090)
A2. Research expenses according to ethical guidelines (Median, IQR)	25674 (0 - 128531)	53236 (566 - 187402)	16346 (0 - 81728)	20326 (0 - 135405)	27504 (0 - 116950)
A3. Non-clinical research expenses (Median, IQR)	10297 (0 - 102396)	11115 (0 - 129884)	14122 (0 - 151192)	7551 (0 - 71005)	16084 (0 - 98165)
A4. Clinical trial expenses (Median, IQR)	0 (0 - 126639)	0 (0 - 200052)	0 (0 - 150826)	0 (0 - 81909)	0 (0 - 70291)
A5. Post-marketing clinical trial expenses (Median, IQR)	0 (0 - 0)	0 (0 - 0)	0 (0 - 0)	0 (0 - 0)	0 (0 - 0)
A6. Failure and infection case reporting expenses (Median, IQR)	0 (0 - 0)	0 (0 - 0)	0 (0 - 0)	0 (0 - 0)	0 (0 - 0)
A7. Post-marketing surveillance expenses (Median, IQR)	424 (0 - 69397)	9913 (0 - 88411)	6731 (0 - 55604)	0 (0 - 88860)	0 (0 - 61234)
A8. Other expense (Median, IQR)	154 (0 - 11798)	2008 (0 - 34532)	49 (0 - 13090)	0 (0 - 8017)	0 (0 - 5766)
**B. Academic research support expenses**					
B1. Scholarship donation (Median, IQR)	65827 (3776 - 657413)	93634 (10194 - 764985)	53236 (6041 - 685268)	66582 (2209 - 618482)	41909 (566 - 603621)
B2. Donation to academic and other societies (Median, IQR)	11327 (755 - 51952)	15102 (3455 - 74847)	15027 (628 - 62843)	9854 (529 - 43268)	5981 (505 - 28336)
B3. General donation (Median, IQR)	15744 (378 - 60126)	23839 (351 - 71679)	14988 (259 - 66828)	12006 (661 - 62297)	12911 (698 - 40512)
B4. Expenses of co-sponsored conferences (Median, IQR)	149045 (40701 - 411133)	172962 (45663 - 415874)	114875 (40720 - 303051)	140879 (31226 - 376486)	167485 (46598 - 401765)
B5. Other expenses (Median, IQR)	27335 (27335 - 27335)	-	-	-	27335 (27335 - 27335)
**C. Lecture, manuscript/writing, and consulting/commissioning fees**					
C1. Lecture fee (Median, IQR)	68791 (17222 - 310657)	91697 (22749 - 376355)	52214 (16812 - 223961)	66324 (9678 - 334541)	80086 (22294 - 314014)
C2. Manuscript writing fee/supervising fee (Median, IQR)	7485 (1430 - 22764)	7691 (1272 - 20902)	8494 (1910 - 21920)	7796 (1999 - 24921)	6525 (1314 - 21283)
C3. Consulting/commissioning fee (Median, IQR)	41059 (7249 - 140237)	51369 (12837 - 171281)	41256 (8676 - 123039)	33250 (8501 - 136756)	23605 (5136 - 111165)
C4. Other expenses (Median, IQR)	-	-	-	-	-
**D. Information provision-related expenses**					
D1. Expenses for conferences (Median, IQR)	57220 (3546 - 430779)	187760 (29831 - 1265187)	29501 (3508 - 314589)	51499 (1289 - 312425)	53574 (5643 - 423755)
D2. Expenses for seminars (Median, IQR)	26857 (6957 - 174048)	70621 (19946 - 302345)	20086 (5720 - 94303)	18589 (2768 - 79640)	29577 (7714 - 147704)
D3. Expenses for provision of literature and other products (Median, IQR)	3364 (0 - 31794)	12210 (0 - 50230)	5452 (0 - 42111)	1694 (0 - 17390)	2295 (0 - 26146)
**E. Other expenses**					
E1. Other expenses (Median, IQR)	19663 (3644 - 82770)	60160 (14015 - 215362)	12772 (3277 - 61213)	8360 (1736 - 45829)	20160 (3550 - 80762)

Median and IQR (interquartile range) was calculated across all companies in the analytic sample for each category and year (including companies with zero payments).

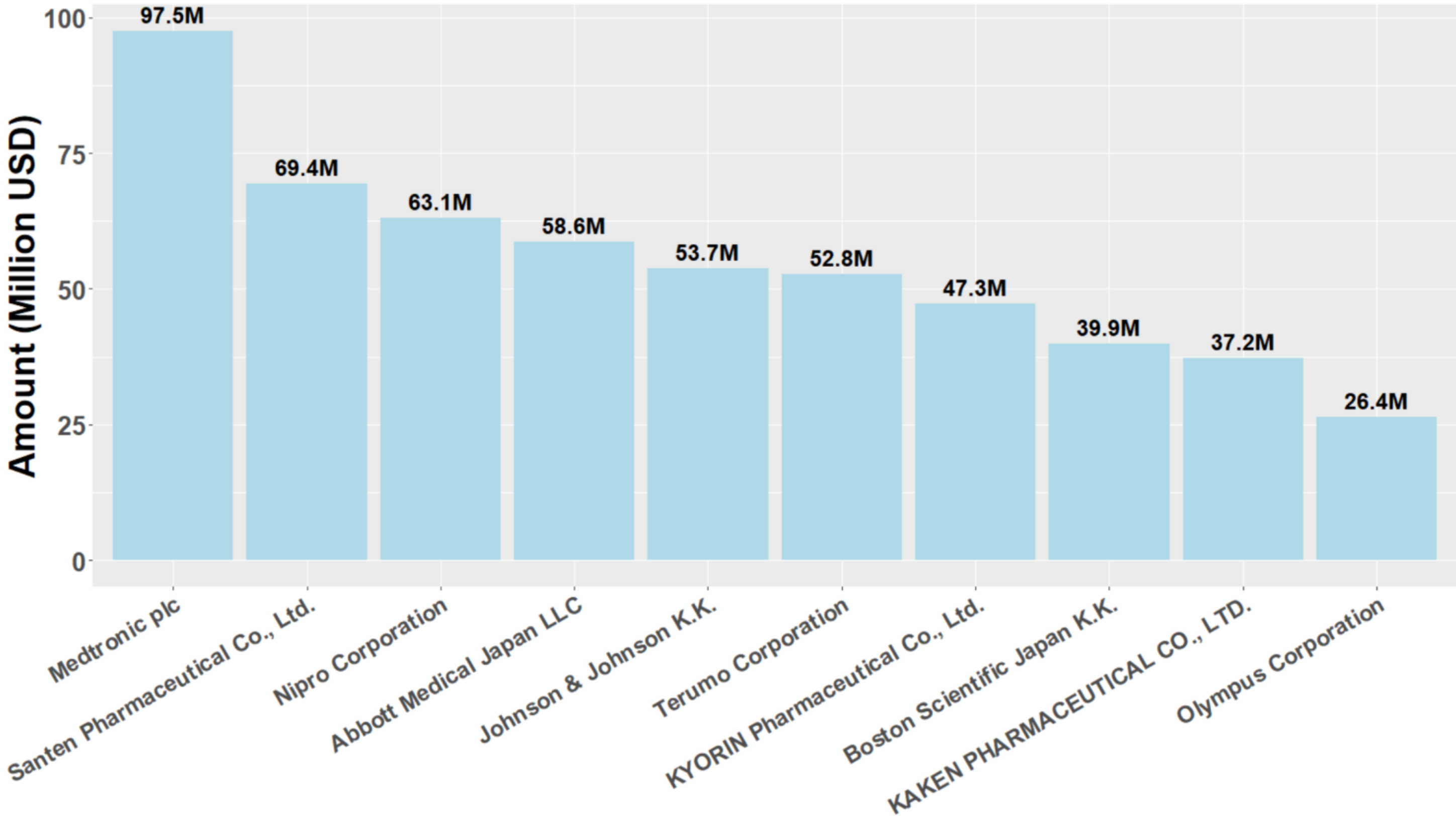

Figure S1. Ranking of Top 10 Medical Device Companies by Total Expenditure from 2019 to 2021 (in Million USD)
